# Impaired autophagy contributes to the aggravated deterioration of osteoarthritis articular cartilage by peroxisome proliferator-activated receptor α deficiency, associated with decreased ERK and Akt activation

**DOI:** 10.1186/s40001-023-01267-4

**Published:** 2023-09-09

**Authors:** Yang Zhou, Li Li, Xiaolei Chen, Qiubo Zhao, Ning Qu, Bing Zhang, Xin Jin, Chun Xia

**Affiliations:** 1 Bone & Joint Research Institute, Zhongshan Hospital, Xiamen University, Xiamen, 361102 China; 2 Department of Orthopedics, Traditional Chinese Medical Hospital of Xinjiang Uygur Autonomous Region, Urumqi, 830000 China; 3 School of Medicine, Xiamen University, Xiamen, 361102 China

**Keywords:** PPARα deficiency, Autophagy, ERK and Akt activation, Mouse model of experimental OA, Human OA chondrocytes

## Abstract

**Background:**

Although the chondroprotection of peroxisome proliferator-activated receptor α (PPARα) activation against osteoarthritis (OA) has been revealed, the regulatory mechanism of PPARα deficiency to aggravate osteoarthritic cartilage deterioration remains unclear. Here, we aimed to investigate whether and how autophagy is involved in OA pathological progression.

**Methods:**

Model of experimental OA was established using destabilization of the medial meniscus in PPARα-KO 129S4/SvJae male mice, followed by histopathological detection of articular cartilage and immunohistochemistry detection of extracellular matrix (ECM) or autophagy-related signal molecules. Meanwhile, human OA chondrocytes obtained from total knee replacement surgery patients with OA were cultured with the pretreatment of IL-1β, followed with the treatment of PPARα agonist WY14643 and the detection of related signal molecules.

**Results:**

PPARα deficiency aggravated cartilage damage with decreased LC3B level in combination with an increase in P62 level, accompanied with reduced p-Akt and p-ERK levels in PPARα-KO mouse model of experimental OA. On the contrary, PPARα activation by WY14643 promoted ECM synthesis in IL-1β-treated human OA chondrocytes, accompanied with increased LC3B-II/I ratio and Beclin 1 level and decreased P62 and Bcl2 levels. Meanwhile, it was observed that activated ERK and Akt by PPARα activation contributed to the enhancement of autophagy and ECM synthesis in human OA chondrocytes.

**Conclusions:**

Impaired autophagy contributed to the aggravated deterioration of osteoarthritis articular cartilage by PPARα deficiency associated with the suppression of ERK and Akt, with an implication that triggering PPARα activation ought to be a potential promising therapeutic target for OA therapy.

**Supplementary Information:**

The online version contains supplementary material available at 10.1186/s40001-023-01267-4.

## Introduction

Osteoarthritis (OA) is a persistent degenerative condition affecting the joints, leading to symptoms such as joint pain and potential difficulties with walking. Joint damage of OA may be partially attributed to extracellular matrix (ECM) degradation, which is secreted by chondrocyte. PPARα, which is part of the class I nuclear receptors, is a member of the ligand-dependent transcription factor superfamily that modifies gene expression through its interaction with the retinoid X receptor [1] or in a DNA binding-independent manner [2] to regulate inflammatory responses [3–5], lipid and lipoprotein metabolism [6], oxidative stress [7] and apoptosis [8]. In recent years, studies have shown that PPARα and autophagy have a regulatory role [9, 10]. It has implicated that PPARα activation has a positive effect on the ECM synthesis of chondrocyte. In osteoarthritic cartilage, the activation of PPARα reduces the inflammatory and destructive reactions [11]. Activation of the PPARα pathway enhances the production of interleukin-1 (IL-1) receptor antagonist in chondrocytes treated with cytokines [12]. However, how PPARα deficiency aggravates osteoarthritic cartilage deterioration remains unclear. Understanding the regulatory mechanism of PPARα deficiency is benefit for OA therapy by PPARα activation.

As a conserved cellular self-digestion process, autophagy could eliminate intracellular unnecessary proteins, pathogens, and damaged organelles to sustain cell survival. OA is one of the human diseases in which autophagy is especially significant in its pathogenesis [13–15]. It has been demonstrated that impaired autophagy could contribute to the pathogenesis of cartilage diseases. The impairment of autophagy caused by the repression of Mfn2 plays a significant role in the development of intervertebral disc degeneration in degenerated human nucleus pulposus tissues [16]. The severity of experimental OA is increased by REDD1 deficiency, which impairs autophagy and mitochondrial biogenesis in articular cartilage [17]. Hence, we hypothesize that the regulation of PPARα deficiency on ECM might be linked to autophagy.

In this study, we observed the histopathological characteristics of articular cartilage and levels of signal molecules related to ECM and autophagy in a mouse model with experimentally induced OA called PPARα-KO 129S4/SvJae. In addition, the regulatory mechanism of PPARα activation by WY14643 linked to autophagy was investigated in IL-1β-treated human OA chondrocytes. Our observations suggest that impaired autophagy contributed to the aggravated deterioration of osteoarthritis articular cartilage by PPARα deficiency, associated with the activation of ERK and Akt, with an implication that triggering PPARα activation ought to be a potential promising therapeutic target for OA therapy.

## Materials and methods

### Antibodies and reagents

Abcam Inc (Cambridge, MA, USA) was the source of the antibodies for PPARα, Collagen II, MMP13, ADAMTS5, and P62. Cell Signaling Technology Inc (Beverly, MA, USA) provided the antibodies for Akt, p-Akt (Ser473), ERK, p-ERK (Thr202/Tyr204), Beclin 1, and ERK inhibitor U0126. Sigma-Aldrich in China (Shanghai, China) supplied the antibody for Aggrecan, Akt inhibitor Triciribine (TCN), and autophagy inhibitor chloroquine diphosphate salt (CQ). Proteintech Group, Inc (Rosemont, IL, USA) provided the antibodies for LC3B and Bcl2. Recombinant human IL-1β was obtained from PeproTech China (Suzhou, China) and β-actin was obtained from Shanghai Abways Biotechnology (Shanghai, China).

### PPARα-KO mouse model of experimental OA

A total of 32 male mice, including 8 ~ 10-week-old Wild Type (WT) mice and 8 ~ 10-week-old PPARα knockout (PPARα-KO) mice, weighing 26 ~ 30 g, were obtained from Jackson Laboratory #003580 (Bar Harbor, ME, USA). Before the experiment, all PPARα-KO mice were identified using PCR (Additional file 2: Figure S1). The study was approved by the Committee on the Ethics of Animal Experiments of Xiamen University. Mice were given intraperitoneal injections of sodium pentobarbital (50 mg/kg) for anesthesia. The right hind knee of each mouse underwent destabilization of the medial meniscus (DMM) to induce the OA model (OA group), while the left knee underwent mocked surgery (Sham group), as previously described [18]. Mice were not euthanized by cervical dislocation until 4 week post-operation, at which point articular cartilage samples were collected for histopathological and immunohistochemical analyses.

### Histopathological assay

The samples that were fixed in 4% paraformaldehyde for 48 h underwent decalcification in 10% EDTA-2Na for 3 weeks and were subsequently paraffin-embedded using the same method explained before [19]. Sagittal plane sections, with a thickness of three micrometers, were taken from the medial side of the joint and stained with Safranin O-Fast green stain. To measure the thickness of the articular cartilage, from the superficial zone to the subchondral bone, in the medial femur condyle and tibial plateau, Image-Pro Plus 6.0 software was utilized by two observers who were blind to the experiment. Subsequently, the OARSI histopathology assessment system was employed to evaluate the level of articular cartilage degeneration [20]. In the OARSI system, a grade of 0 indicates normal cartilage, while a higher grade (ranging from 1 to 24) indicates a greater level of biological cartilage degeneration.

### Immunohistochemistry assay

As per the manufacturer's instruction (MAIXIN.BIO, Fuzhou, China), three-micrometer-thick sections were incubated overnight at 4 °C with primary antibodies: Aggrecan (1:600), Collagen (1:100), LC3B (1:100), P62 (1:100), p-Akt (1:50), and p-ERK (1:50) dilutions. After that, secondary antibodies were applied. The immunohistochemical reaction was visualized using diaminobenzidine and counterstained with haematoxylin. Photomicrographs were captured with OLYMPUS BX41 microscope equipped with a digital camera. The positive chondrocytes expressing LC3B, P62, p-Akt, and p-ERK were counted semi-automatically using Image-Pro Plus 6.0 Software, and the area was measured using Image J Software. Subsequently, the data were analyzed with Graph-Pad Prism version 5, as previously described [19].

### Human OA chondrocyte isolation and culture

Once the Ethics Committee of Medical School, Xiamen University granted approval for this project and all patients involved provided informed consent, human OA cartilage was obtained from the anonymous waste material of 12 OA patients who were undergoing total knee replacement surgery (Additional file 1: Table S1). Prior to surgery, the OA patients had refrained from taking any non-steroidal anti-inflammatory drugs or steroids for a minimum of 2 weeks, and had not received any intra-articular injection for at least 1 month. The explants were then stored in liquid nitrogen for chondrocyte culture, as mentioned in previous literature [21, 22], primary chondrocytes were grown in DMEM (10% fetal bovine serum) until they reached 80% confluence, and then were used to seed 60-mm Petri dishes or 96-well plates. To maintain an inflammatory state in human OA chondrocytes, chondrocytes obtained from OA patients were consistently exposed to IL-1β for the following experiments.

### Western blotting analysis

As previously described, protein extracts were obtained by lysing and collecting cells through centrifugation [21, 23], the latter were subjected to SDS–PAGE (8–12%) and transferred to a PVDF membrane (GE Healthcare, Herfordshire, UK). The membrane was subjected to overnight incubation at 4 °C with different primary antibodies as needed, and was then treated with the appropriate secondary antibodies at room temperature for 1 to 2 h. The reactivity of the antibodies was detected using an enhanced chemiluminescence detection kit from Pierce, Rockford, IL, USA.

### Transmission electron microscopy

The cells were collected and then centrifuged at 2000 × *g* for 15 min at 4 °C. Afterwards, they were fixed for 2 h at 4 °C in 2.5% glutaraldehyde in 0.1 M PBS (PH7.4) following the same procedure as described before [21, 24]. The samples underwent dehydration and were then embedded in Embed-812 resin. To obtain 70 nm sections, an ultramicrotome (Leica EM UC7, LEICA, Shanghai, China) was used, and these sections were stained with uranyl acetate and lead citrate. The transmission electron microscope (Tecnai G2 Spirit BioTWIN, FEI Company, Hillsboro, Oregon, USA) was utilized to observe autophagic vacuoles.

### Dimethylmethylene blue assay

Glycosaminoglycan (GAG) contents in culture medium were measured using dimethylmethylene blue assay. Cells were harvested by centrifugation to extract DNA. The culture medium was treated with 2 mg/ml papain in an HEPES buffered saline solution (HBS) for a duration of 12 h at a temperature of 60 °C. A standard of shark chondroitin sulfate from Sigma-Aldrich (St. Louis, MO, USA) was used, with a range of 0 to 70 ng. To each well of a 96-well plate, 20 μl of a papain solution, as well as each standard and sample dilution in triplicate, were added sequentially. Following this, 200 μl of a DMMB solution was added to each well and the plate was read at a wavelength of 630 nm using a spectrophotometer within a time frame of 10 min. The amount of GAGs was adjusted based on the DNA content, which was measured using an infinite M200 instrument (Tecan, Switzerland)[21, 25].

### Statistical analysis

The mean ± SD of at least three independent experiments in each cell experiment and 6 independent samples in each group of animal experiment were presented as the data. The statistical significance between the groups was assessed using the *t* test for comparisons between two groups and one-way analysis of variance (ANOVA) for comparisons among groups or more. Tukey's post hoc tests were conducted following the analysis, using GraphPad Prism version 5 (GraphPad Software, Inc, San Diego, CA, USA). A *p* value less than 0.05 was considered significant.

## Results

### PPARα deficiency aggravated articular cartilage damage in PPARα-KO mouse model of experimental OA

**Fig. 1:**
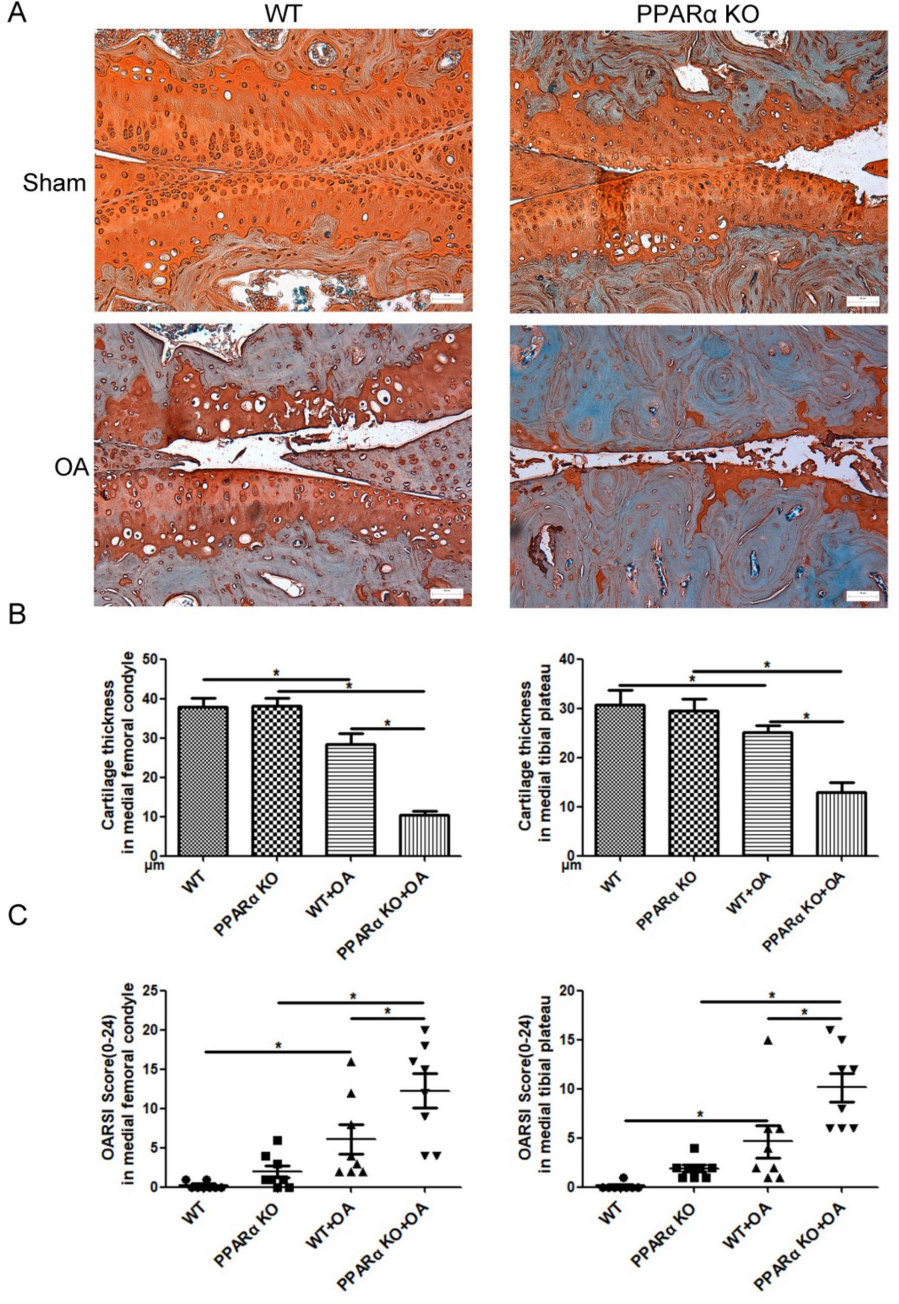
Aggravated cartilage damage in PPARα-KO mouse model of experimental OA by DMM. **A** Sections in different treated groups were examined using Safranin O-Fast green stain (original magnification × 20); **B** graph shows the change in articular cartilage thickness in medial femur condyle and tibial plateau. Data are mean ± SD of eight joints per group (*p < 0.05); **C** graphs show the histopathological assay performed by OARSI histopathology assessment system in medial femur condyle and tibial plateau. Data are mean ± SD of eight joints per group (*p < 0.05)

**Fig. 2:**
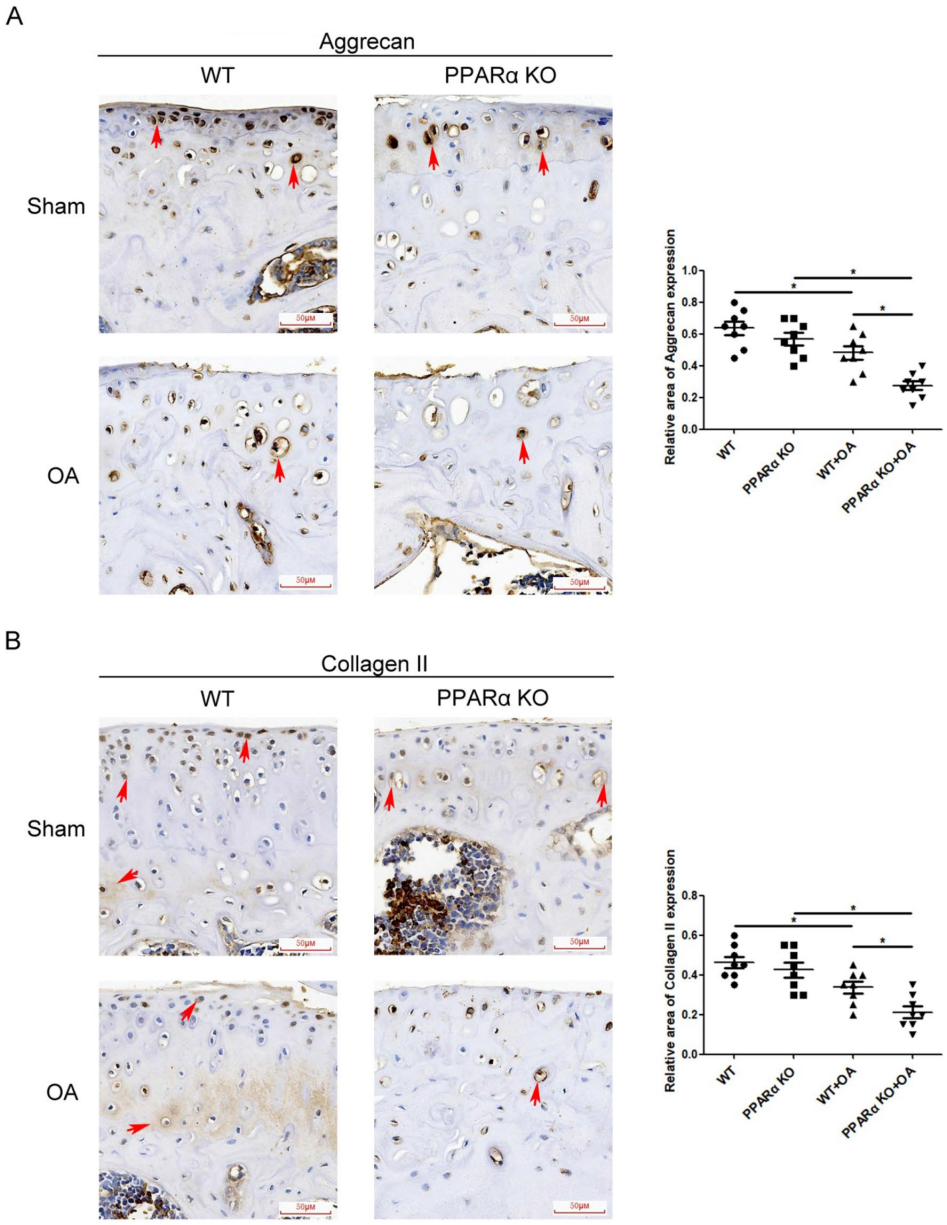
Expression of ECM synthesis biomarkers in cartilage of PPARα-KO mouse model of experimental OA. Sections in different treated groups were examined using immunohistochemistry assay; **A** representative image from mice of different treated groups expressing Aggrecan (original magnification × 40). Graph shows the relative area expressing Aggrecan. Data are mean ± SD of 24 fields of vision per group (*p < 0.05); **B** representative images from mice of different treated groups expressing Collagen II (original magnification × 40). Graph shows the relative area expressing Collagen II. Data are mean ± SD of 24 fields of vision per group (*p < 0.05)

Safranin O-Fast green staining was used to identify the histological features of knee joints in PPARα-KO mice following DMM. The image shown in Fig. 1A demonstrated the typical structure of the matrix and a proper distribution of cells in the WT sample of the Sham group. Conversely, the PPARα-KO sample from the Sham group showed a lack of continuity in the superficial zone of the articular cartilage and a depletion of matrix (indicated by reduced Safranin O staining) in the upper one-third of the cartilage. However, there was no difference in cartilage thickness (femoral condyle and medial tibial plateau) and OARSI Score (0–24) between WT and PPAR-KO samples of Sham group (Fig. 1B, C). Compared with WT sample of OA group, there were heavy loss of cartilage matrix and cells at superficial and middle layer in PPARα-KO sample of OA group, concomitant with a significant difference of cartilage thickness and OARSI Score between WT and PPARα-KO sample in OA group (Fig. 1B, C, *p < 0.05). In the meantime, we observed a noteworthy decrease in the thickness of cartilage and an increase in the OARSI score in the medial femoral condyle and medial tibial plateau of the OA group's WT sample when compared to the Sham group's WT sample. In the PPARα-KO sample of the OA group, there was a significant decrease in cartilage thickness and an increase in OARSI score in the medial femoral condyle and medial tibial plateau compared to the PPARα-KO sample of the Sham group (Fig. 1B, C, *p < 0.05). In addition, there was no difference in the area of Aggrecan and Collagen II expression between the WT and PPARα-KO samples in the Sham group, but a significant difference was observed between the WT and PPARα-KO sample of the OA group (indicated by the red arrows, Fig. 2A, B, *p < 0.05). Furthermore, the levels of Aggrecan and Collagen II in the WT sample of the OA group were lower than that in the WT sample of the Sham group. Comparing the PPARα-KO sample of the Sham group, there was a significant decrease in Aggrecan and Collagen II levels in the PPARα-KO sample of the OA group (Fig. 2A, B, *p < 0.05). Overall, the data indicate that PPARα deficiency worsens articular cartilage damage, leading to reduced cellularity, Aggrecan, and Collagen II levels in the experimental OA mouse model, but not in the Sham group.

### PPARα deficiency decreased LC3B-II/I ratio in combination with an increase in the P62 level, with reduced p-ERK and p-Akt levels in PPARα-KO mouse of experimental OA

**Fig. 3:**
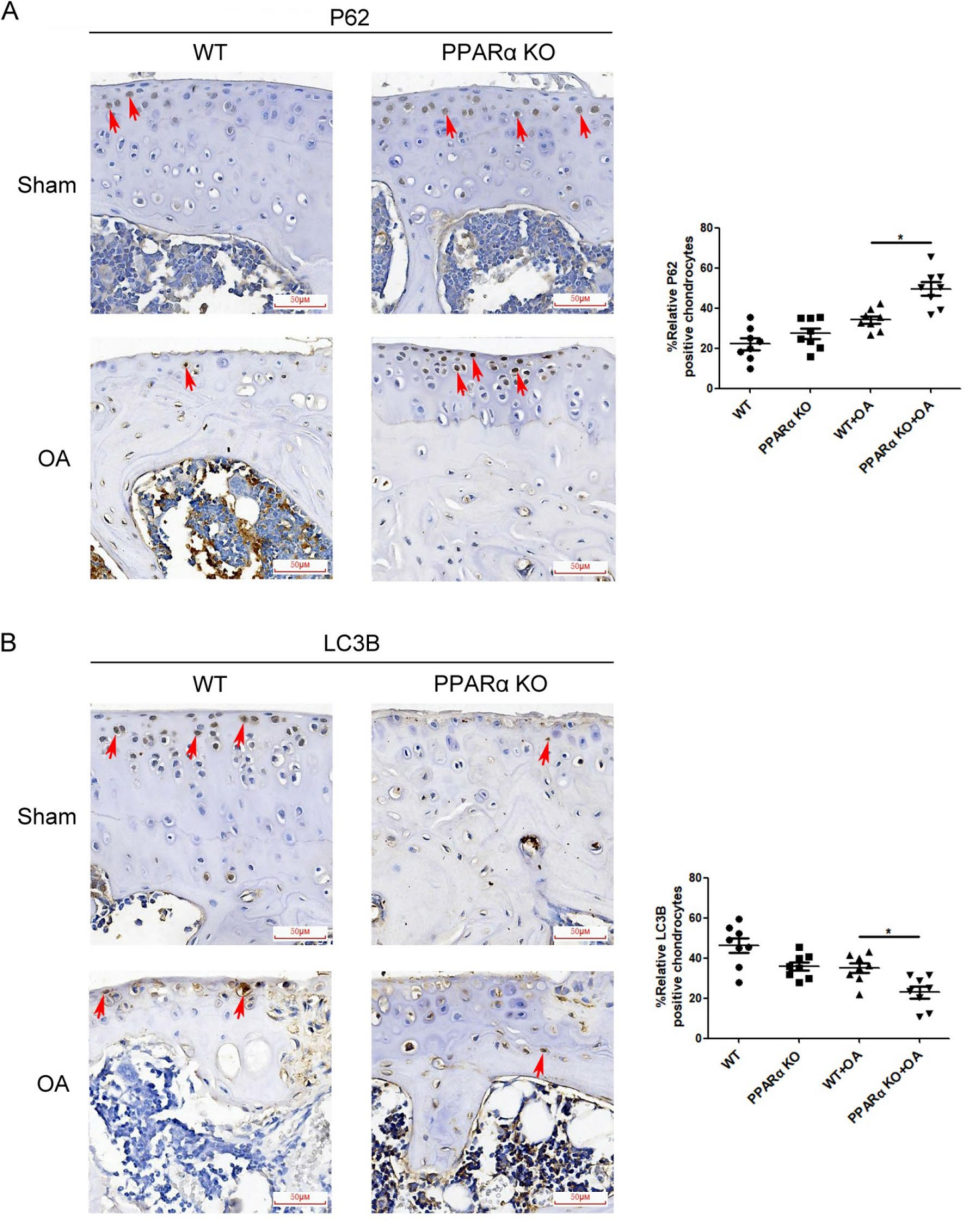
Expression of autophagy biomarkers in cartilage of PPARα-KO mouse model of experimental OA. Sections in different treated groups were examined using immunohistochemistry assay. **A** Representative image from mice of different treated groups expressing P62 (original magnification × 40). Graph shows the percentage of positive chondrocytes expressing P62. Data are mean ± SD of 500 cells per group (*p < 0.05); **B** representative images from mice of different treated groups expressing LC3B (original magnification × 40). Graph shows the percentage of positive chondrocytes expressing P62. Data are mean ± SD of 500 cells per group(*p < 0.05)

**Fig. 4:**
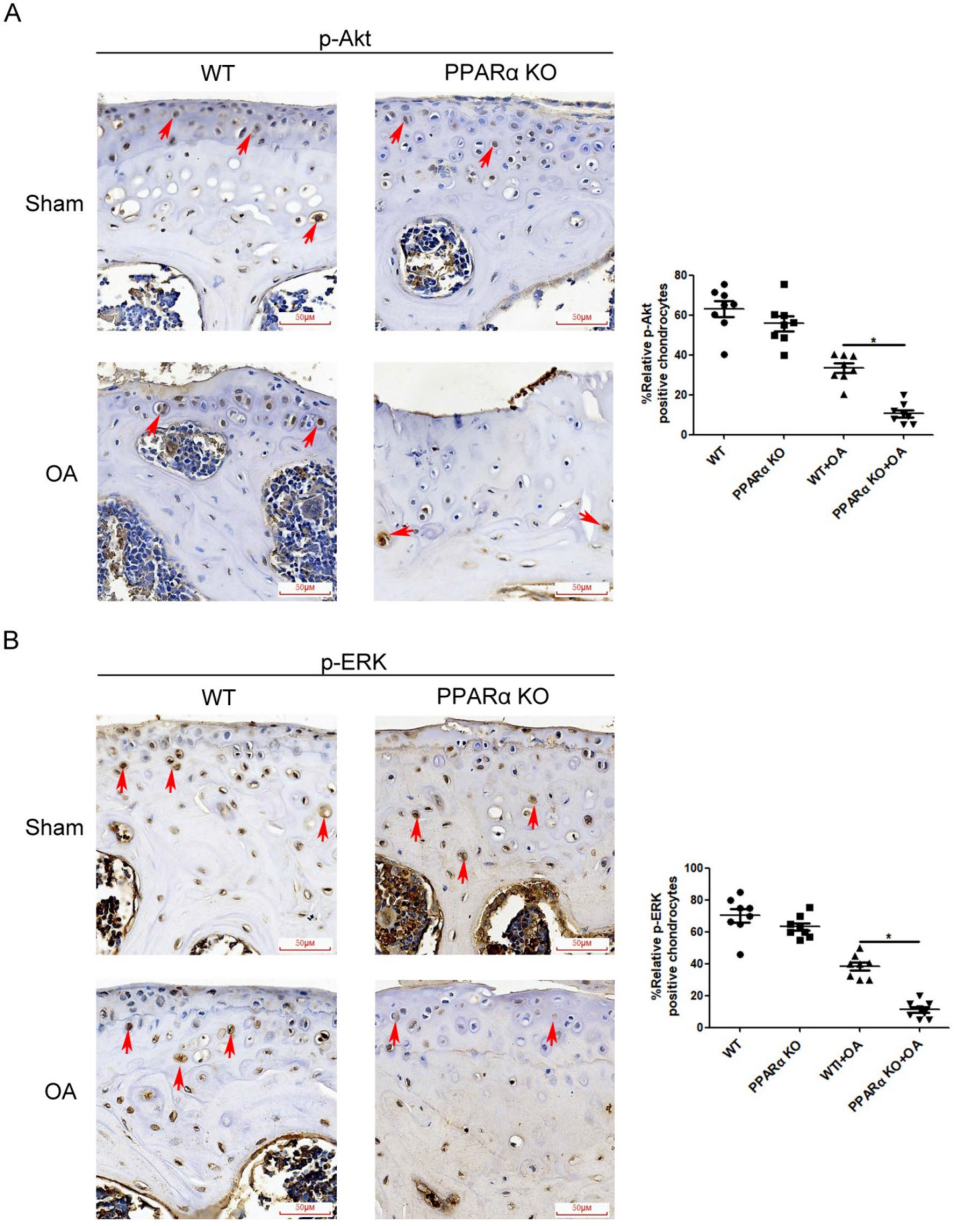
Expression of p-ERK and p-Akt in cartilage of PPARα-KO mouse model of experimental OA. Sections in different treated groups were examined using immunohistochemistry assay. **A** Representative image from mice of different treated groups expressing p-ERK (original magnification × 40). Graph shows the percentage of positive chondrocytes expressing p-ERK. Data are mean ± SD of 500 cells per group (*p < 0.05); **B** representative images from mice of different treated groups expressing LC3B (original magnification × 40). Graph shows the percentage of positive chondrocytes expressing P62. Data are mean ± SD of 500 cells per group (*p < 0.05)

In Sham group, compared with WT sample, chondrocytes in PPARα-KO sample displayed a higher P62 level in combination with a lower LC3B level, but without significant difference (Fig. 3). In OA group, there was a significant increase in the P62 level with a decrease in LC3B level between PPARα-KO and WT sample (indicated by the red arrows, Fig. 3, *p < 0.05). In addition, in Sham group, the phosphorylation of ERK (p-ERK) and Akt (p-Akt) in chondrocytes of PPARα-KO sample seemed to be lower than that of WT sample, but without significant difference (indicated by the red arrows, Fig. 4). In OA group, there were significant differences in p-ERK and p-Akt levels between PPARα-KO and WT sample, expressing reduced p-ERK and p-Akt levels in PPARα-KO sample (indicated by the red arrows, Fig. 4, *p < 0.05). Therefore, the data showed that PPARα deficiency decreased LC3B level in combination with an increase in the P62 level, accompanied with reduced the levels of p-ERK and p-Akt in PPARα-KO mice with OA.

### PPARα activation by WY14643 enhanced Aggrecan, and Collagen II levels accompanied with the alteration of LC3B-II/I ratio, Beclin1, P62, and Bcl2 levels in IL-1β-treated human OA chondrocytes.

**Fig. 5:**
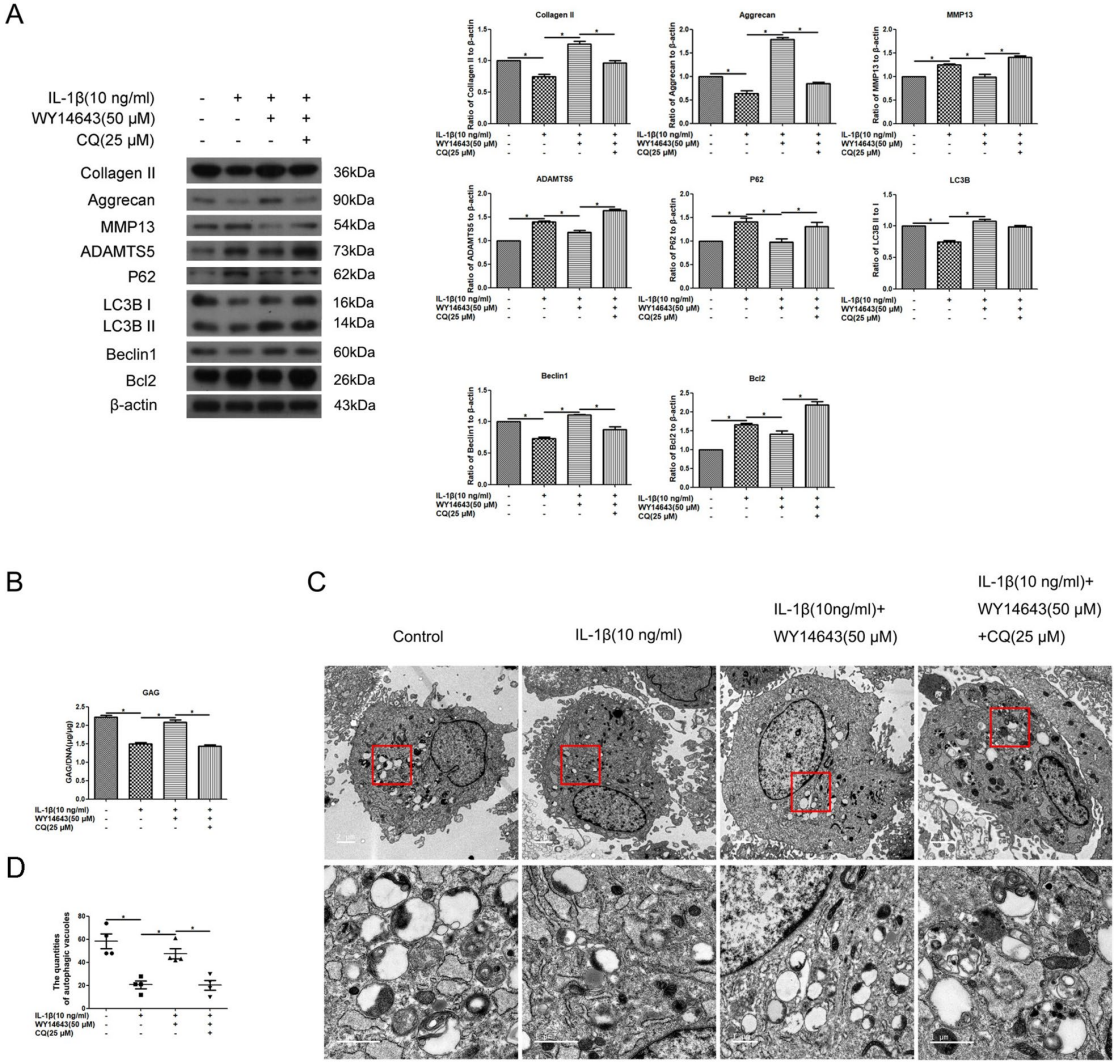
Effect of PPARα activation by WY14643 on the expression of ECM and autophagy-related signal molecules in IL-1β-treated human OA chondrocytes. Cells were pretreated with WY14643 (50 μM), CQ (10 μM) or WY14643 (50 μM) + CQ (10 μM) for 2 h, respectively, and were then co-treated with IL-1β (10 ng/ml) for 22 h. **A** Expression of Collagen II, Aggrecan, MMP13, ADAMTS5, P62, LC3B, Beclin1, Bcl2, and β-actin protein levels were detected via western blotting; **B** GAG levels were measured via Dimethylmethylene blue assay; **C** autophagic vacuoles were observed under a transmission electron microscope; **D** quantities of autophagic vacuoles. The data are representative of three independent experiments (*p < 0.05)

Figure 5A illustrates that Collagen II and Aggrecan levels increased, while MMP13 and ADAMTS5 levels decreased due to PPARα activation by WY14643 (*p < 0.05, compared to the IL-1β-treated group). In addition, activated PPARα resulted in an increase in the LC3B-II/I ratio and a decrease in the P62 level, along with an increase in the Beclin1 level and a decrease in the Bcl2 level (Fig. 5A, *p < 0.05, compared to the IL-1β-treated group). Furthermore, the addition of the autophagy inhibitor CQ partially attenuated the effect of WY14643, leading to a decrease in the Beclin 1 level and an increase in the P62 and Bcl2 levels, without significant changes in the LC3BII/I ratio (Fig. 5A, *p < 0.05, compared to the IL-1β + WY14643-treated group). In Fig. 5B, it can be observed that GAG levels in the culture medium of IL-1β + WY14643 human OA chondrocytes were higher than those in the IL-1β-treated group; however, CQ diminished the effect of activated PPARα by WY14643 on GAG as compared to the IL-1β + WY14643-treated group (*p < 0.05). Using a transmission electronic microscope, it was observed that IL-1β + WY14643-treated group had an increased number of autophagic vacuoles compared to the IL-1β-treated group, whereas accumulation of autophagic vacuoles could be observed in the IL-1β + WY14643 + CQ-treated group (Fig. 5C). In conclusion, these findings suggest that PPARα activation by WY14643 enhances Aggrecan and Collagen II levels and alters the levels of Beclin 1, P62, and Bcl2 in IL-1β-treated human OA chondrocytes.

### PPARα activation by WY14643 elevated the phosphorylation of ERK and Akt in IL-1β-treated human OA chondrocytes

**Fig. 6:**
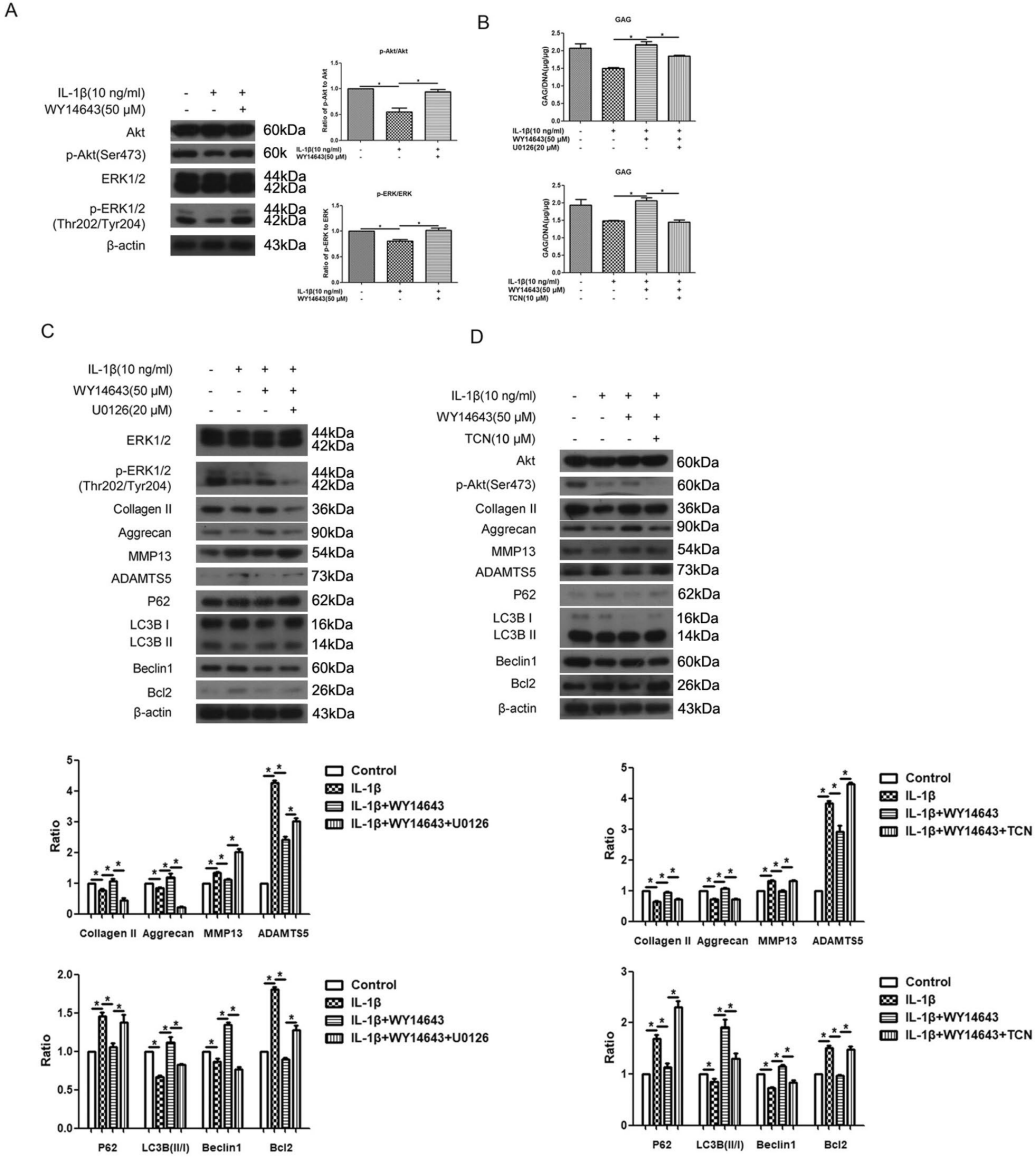
Expression of p-ERK and p-Akt in IL-1β-treated human OA chondrocytes. **A** Cells pretreated WY14643 (50 μM) for 2 h were co-treated with IL-1β (10 ng/ml) for 22 h. The expression of Akt, p-Akt, ERK, p-ERK, and β-actin protein levels were detected via western blotting; **B** cells were pretreated with WY14643 (50 μM), U0126 (20 μM), WY14643 (50 μM) + U0126 (20 μM) and WY14643 (50 μM) + TCN (10 μM) for 2 h, respectively, and were then co-treated with IL-1β (10 ng/ml) for 22 h. GAG levels were measured via Dimethylmethylene blue assay; **C** cells were pretreated with WY14643 (50 μM), U0126 (20 μM) or WY14643 (50 μM) + U0126 (20 μM) for 2 h, respectively, and were then co-treated with IL-1β (10 ng/ml) for 22 h. The expression of ERK, p-ERK, Collagen II, Aggrecan, MMP13, ADAMTS5, P62, LC3B, Beclin1, Bcl2, and β-actin protein levels were detected via western blotting; **D** cells were pretreated with WY14643 (50 μM), TCN (20 μM) or WY14643 (50 μM) + TCN (10 μM) for 2 h, respectively, and were then co-treated with IL-1β (10 ng/ml) for 22 h. The expression of Akt, p-Akt, Collagen II, Aggrecan, MMP13, ADAMTS5, P62, LC3B, Beclin1, Bcl2, and β-actin protein levels were detected via western blotting. The data are representative of three independent experiments (*p < 0.05)

Activated PPARα by WY14643 in IL-1β-treated human OA chondrocytes increased the phosphorylation of ERK and Akt, as well as GAGs levels (Fig. 6A, *p < 0.05, vs IL-1β-treated group). The addition of ERK inhibitor U0126 or Akt inhibitor TCN partially reduced the upregulated p-ERK and p-Akt, as well as increased GAGs levels by activated PPARα (Fig. 6B–D, *p < 0.05, vs IL-1β + WY14643-treated group). Moreover, both U0126 and TCN reversed the effect of activated PPARα on ECM and autophagy-related biomarkers (Collagen II, Aggrecan, MMP13, ADAMTS5, LC3B, P62, Beclin1, Bcl2) (Fig. 6C, and D, *p < 0.05). Consequently, PPARα activation by WY14643 increased the phosphorylation of ERK and Akt in IL-1β-treated human OA chondrocytes.

## Discussion

The primary finding of this study is that the absence of PPARα in mice increased the severity of experimental OA. In comparison with the control group, the PPARα-KO mouse model experienced more significant deterioration of the articular cartilage, resulting in reduced cellularity, Aggrecan, and Collagen II levels. Conversely, the use of WY14643, a PPARα activator, was found to increase the levels of Aggrecan and Collagen II in human OA chondrocytes treated with IL-1β. Taking into consideration the advantages and disadvantages of these results, it emphasizes the crucial role of PPARα in the progression of OA.

It has been suggested that PPARα activation provides defense against inflammatory reactions. For instance, the activation of PPARs, namely, α, β/δ, and γ, inhibits collagen production induced by TGF-β1 and adjusts the balance between TIMP-1 and MMPs in chondrocytes cultured in three dimensions [26]. In chondrocytes, the expression of TGF-β and MMP-9 is reduced by PPARα in response to AGE stimulation [27]. The activation of PPARα pathway enhances the production of interleukin-1β receptor antagonist in cytokine-treated chondrocytes [12]. The main function of PPAR is to directly or indirectly promote the inactivation of NF-κB during inflammatory reactions [28]. In agreement with these studies, our findings in human OA chondrocytes also demonstrated that PPARα activation by WY14643 enhanced Aggrecan, and Collagen II levels. Because of the protection by PPARα activation, there were some signs of cartilage damage in articular cartilage in Sham PPARα KO mice without DMM operation, such as the loss of ECM and aberrant cellular architecture.

Mounting evidence supports that autophagy is an important regulator of OA pathogenesis [14, 29, 30]. Another finding of this study is that PPARα deficiency impaired autophagy in chondrocyte of experimental OA in mice. Compared to WT mice, the PPARα-KO mouse model of experimental OA induced by DMM showed a decrease in autophagy, which was observed through a reduction in LC3B level and an increase in P62 level. Furthermore, our previous research has demonstrated that when WY14643 activates PPARα, it results in an elevated LC3B-II/I ratio and a reduction in P62 levels in mouse chondrocytes treated with LPS and in an OA model [19], and we demonstrate that in IL-1β-treated human OA chondrocytes, the activation of PPARα resulted in an elevation of the Beclin1 level and a reduction in the Bcl2 level. This suggests that PPARα activation also enhances autophagy by disrupting the Beclin1–Bcl2 complex. It is widely recognized that Bcl-2/Bcl-XL can hinder autophagy by competing with Beclin-1 [23, 31]. This discovery provided more insight into the mechanisms that controlled autophagy in chondrocytes and emphasized the significance of PPARα in regulating autophagy through various pathways.

PPARα activation by its ligands could activate ERK in a variety of different cell types [32, 33]. Meanwhile, activating ERK by extracellular factors, such as cisplatin [34] and Platycodin D [35] could promote autophagy in cancer cells. Thus, it is believed that ERK activation associated with PPARα might positively regulate autophagy. Here, PPARα–ERK pathway partially contributed to autophagy enhancement in human OA chondrocyte. However, in contrast to the observation made by Ravingerová et al. in WY14643-treated rats' ischaemic myocardium, we noticed an increase in Akt phosphorylation in human OA chondrocytes treated with WY14643 [36]. Combining our findings that the Akt inhibitor TCN reversed the impact of activated PPARα by WY14643 on biomarkers related to ECM and autophagy, it indicates that the PPARα–Akt pathway may enhance autophagy to stimulate ECM synthesis in human OA chondrocytes. Despite Akt typically being known for its negative role in regulating autophagy, some studies have suggested the opposite. Lu et al. reported that rasfonin boosts autophagy by simultaneously reducing mTORC1 signaling and increasing Akt activity through the glycolytic pathway [37]; Dou et al. testify that the class IA phosphatidylinositol 3-kinase p110-β subunit acts upstream of Akt to trigger autophagy [38]. Akt might execute different or opposite functions in autophagy regulation, presumably due to different cell contexts and metabolic states.

A limitation to this study is the lack of precisely targeting PPARα activation in articular chondrocyte in mouse model of experimental OA. Another one is that we did not investigate the effect of PPARα agonist combined with autophagy activator on cartilage ECM synthesis in animal model of experimental OA. Last one is that we did not provide new insights into the molecular mechanism, except for the relationship between PPARα and autophagy.

## Conclusions

**Fig. 7:**
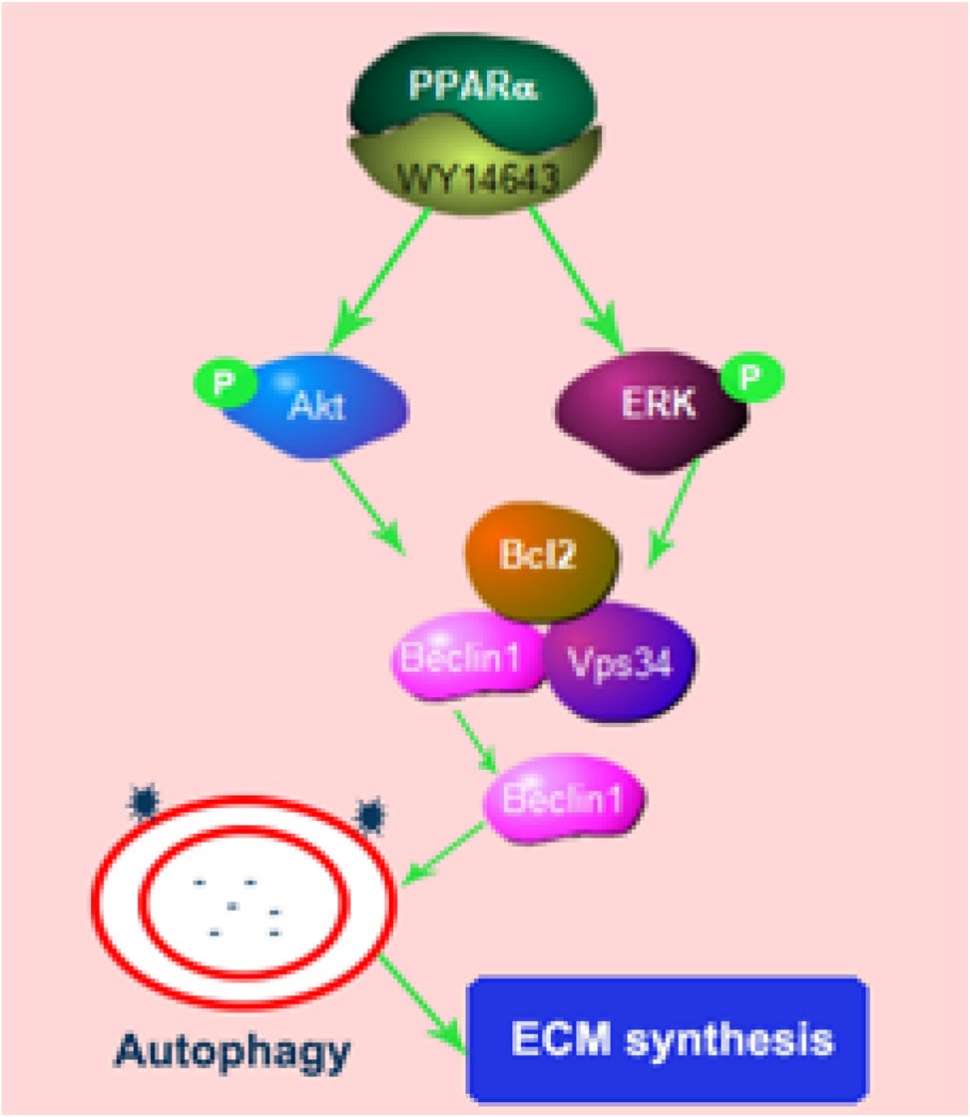
Schematic depiction of proposed mechanism by which PPARα-driven autophagy confers protection against OA in human OA chondrocytes and PPARα-KO mouse model of experimental OA

The deterioration of cartilage with impaired autophagy was further exacerbated by the absence of PPARα, as confirmed by our findings. The activation of PPARα with WY14643 stimulated autophagy by engaging ERK and Akt, leading to an increase in LC3B-II/I ratio, a decrease in P62 level, and the separation of Beclin1 from the Beclin1–Bcl2 complex. This ultimately enhanced the synthesis of ECM in chondrocytes affected by OA (Fig. 7). Consequently, initiating the activation of PPARα should be considered as a potentially effective target for treating OA.

### Supplementary Information

**Additional file 1: Table S1.** Patient information for individuals undergoing total knee replacement surgery.

**Additional file 2: Figure S1.** PCR for PPARα-KO mice Gene identification.

## Data Availability

The data sets used and/or analyzed during the current study are available from the corresponding author upon reasonable request.
