# Supplementary material for: Impaired autophagy contributes to the aggravated deterioration of osteoarthritis articular cartilage by peroxisome proliferator-activated receptor α deficiency, associated with decreased ERK and Akt activation
Source: Eur J Med Res. 2023 Sep 9;28:332. doi: 10.1186/s40001-023-01267-4 (PMC10492277; doi:10.1186/s40001-023-01267-4)
Supplement: Supplementary file 1 — Additional file 1: Table S1. Patient information for individuals undergoing total knee replacement surgery. [file 40001_2023_1267_MOESM1_ESM.docx]

| Age（Year） | Case | Sex | | Duration of OA（year） | | K.L Image Criterion | | Pro-Treatment Arthroscopy |
| --- | --- | --- | --- | --- | --- | --- | --- | --- |
|  |  | M | F | ≤3 | ＞3 | III | IV |  |
| 60- | 12 | 2 | 10 | 5 | 7 | 2 | 10 | 0 |
