# Supplementary figures and images for: Impaired autophagy contributes to the aggravated deterioration of osteoarthritis articular cartilage by peroxisome proliferator-activated receptor α deficiency, associated with decreased ERK and Akt activation
Source: Eur J Med Res. 2023 Sep 9;28:332. doi: 10.1186/s40001-023-01267-4 (PMC10492277; doi:10.1186/s40001-023-01267-4)

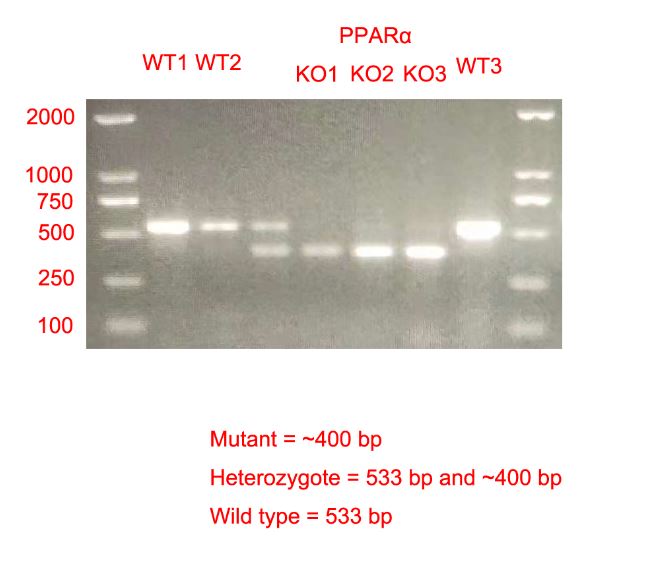

Supplement: Supplementary file 2 — Additional file 2: Figure S1. PCR for PPARα-KO mice Gene identification. [file 40001_2023_1267_MOESM2_ESM.jpg]
